# Feature engineering, parameter optimization, and interpretable modeling for accelerometer-based behavior recognition in aviary laying hens

**DOI:** 10.1016/j.psj.2026.107539

**Published:** 2026-07-30

**Authors:** Xiao Yang, Luwei Nie, Qunpeng Niu, Xiaoqiang Li, Juncheng Ma, Chaoyuan Wang

**Affiliations:** a College of Water Resources and Intelligence Engineering, China Agricultural University, Beijing, 100083, China; b Key Laboratory of Agricultural Engineering in Structure and Environment, Ministry of Agriculture and Rural Affairs, Beijing, 100083, China; c College of Animal Science, Ningxia University, Yinchuan, 750021, China

**Keywords:** Laying hen, Behavior, Acceleration, Machine learning, Deep learning

## Abstract

Behavior monitoring is essential for assessing the health and welfare of laying hens. As the dominant trend in global egg production, aviary systems introduce considerable challenges for behavior recognition. Existing studies remain fragmented and lack standardized analytical guidelines for sliding window configuration, feature selection, and model choice, making it difficult to compare results across studies and to develop robust, generalizable systems. Therefore, this study proposes a comprehensive framework for accelerometer-based behavior recognition. A total of 25 Hy-Line laying hens were reared in an aviary system at the age of 65 weeks. Ten light-weight accelerometers were attached to the wings of selected birds to collect behavioral data at a sampling frequency of 15 Hz. Resting, drinking, feeding, walking, and flying behaviors were investigated in this study. Hundreds of features were automatically extracted using scalable hypothesis testing, and the effects of feature quantity on model performance was evaluated. Five machine learning (ML) models and four deep learning (DL) models were compared under four sliding window sizes (1 s, 2 s, 3 s, and 5 s) and five overlap ratios (0%, 25%, 50%, 70%, and 90%), to determine the optimal configuration. Additionally, interpretability analysis was conducted for the best-performing ML model. The results indicated that Extreme Gradient Boosting (XGB) and one-dimensional Convolutional Neural Network (1D-CNN) achieved the best performance among ML and DL models, respectively. XGB achieved an overall accuracy of 0.985 and an F1 score of 0.987, while 1D-CNN achieved an accuracy of 0.985 and an F1 score of 0.985. The optimal configuration was a 3-s sliding window with 50% overlap. Model interpretation revealed that acceleration variability, amplitude, and energy-related features were the most influential for behavior discrimination, with significant interactions among features. This study provides practical insights for developing acceleration-based behavior classification technologies and supports precision poultry management.

## Introduction

Behaviors are important indicators of the health status and welfare conditions of laying hens (Ma et al., 2026). In modern commercial egg production, aviary systems are increasingly adopted due to their capability of allowing birds to move freely within a three-dimensional space and express natural behaviors (Jeon et al., 2025; Ma et al., 2026). While such systems improve animal welfare, they also pose significant challenges for behavior monitoring because of diverse behavior types, dynamic behavioral patterns, and complex environmental conditions (Louton et al., 2016). Therefore, accurate and automated recognition of laying hen behaviors is essential not only for precision management of poultry health and welfare, but also for aviary system design and optimization (Ma et al., 2026).

Accelerometer-based sensing has emerged as a promising solution for behavior recognition of aviary birds, offering advantages such as robustness to environmental conditions and continuous data acquisition at the individual level (Sakib et al., 2025; Yang et al., 2025). Many studies have applied machine learning and deep learning approaches to classify behaviors of various livestock species such as cattle, pig, and sheep, demonstrating the feasibility of using accelerations signals for behavior recognition in poultry (Lin et al., 2025; Ramos et al., 2026; Wang et al., 2025). However, few studies have focused on laying hens in aviary systems, where behaviors exhibit frequent transitions, higher level of signal noise, and less distinct movement pattern, making classification considerably more challenging. Therefore, a deeper understanding of how acceleration data represent laying hen behaviors, along with comprehensive extraction of informative signal features, is essential for achieving reliable model performance. At present, there is a lack of standardized guidelines for parameter selection in such analyses, limiting the cross-sectional and longitudinal comparisons among studies.

In existing studies, feature engineering is often limited to a small set of handcrafted features derived from prior knowledge, and the impact of feature quantity on model performance is rarely investigated (Mei et al., 2023). It remains unclear whether the selected features are sufficiently representative or whether additional informative features are overlooked. In addition, sliding window parameters, including window size and overlap ratio, play a critical role in defining the temporal structure of input data, yet their effects are often not comprehensively evaluated. Meanwhile, deep learning models are increasingly applied due to their strong performance in behavioral recognition tasks for other species (Yang et al., 2024); however, systematic comparisons between machine learning and deep learning under unified experimental settings remain limited. Their respective advantages in this task are not fully understood. Furthermore, the interpretability of behavior recognition models is often neglected. Most studies focus primarily on classification accuracy without explaining how features contribute to predictions, which restricts biological understanding and limits practical applications. In addition, interpretability can provide valuable insights for guiding efficient model optimization, rather than relying solely on increasing model complexity.

To address these gaps, this study proposes a comprehensive framework for accelerometer-based behavior recognition in aviary laying hens. High-dimensional feature vectors are automatically extracted, and the effects of feature quantity on model performance was evaluated. Multiple widely used machine and deep learning models were compared under four sliding window sizes and five overlap ratios to identify the optimal parameter configurations. Furthermore, interpretation of the best-performing machine learning model was conducted to provide insights into global feature importance, behavior-specific characteristics, and feature interactions.

## Materials and methods

### Animals, housing, and management

**Fig. 1:**
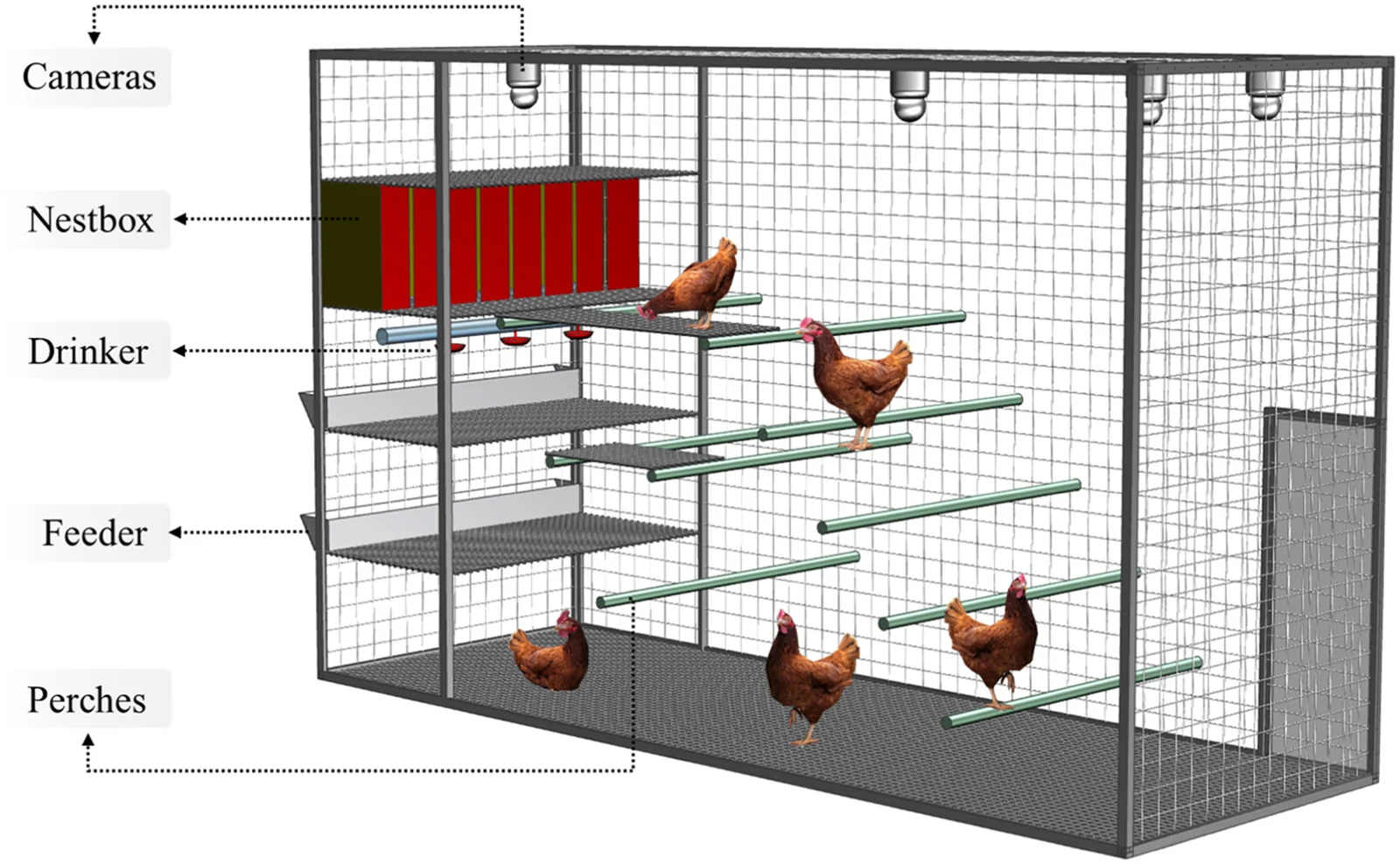
Aviary system design in this study.

**Fig. 2:**
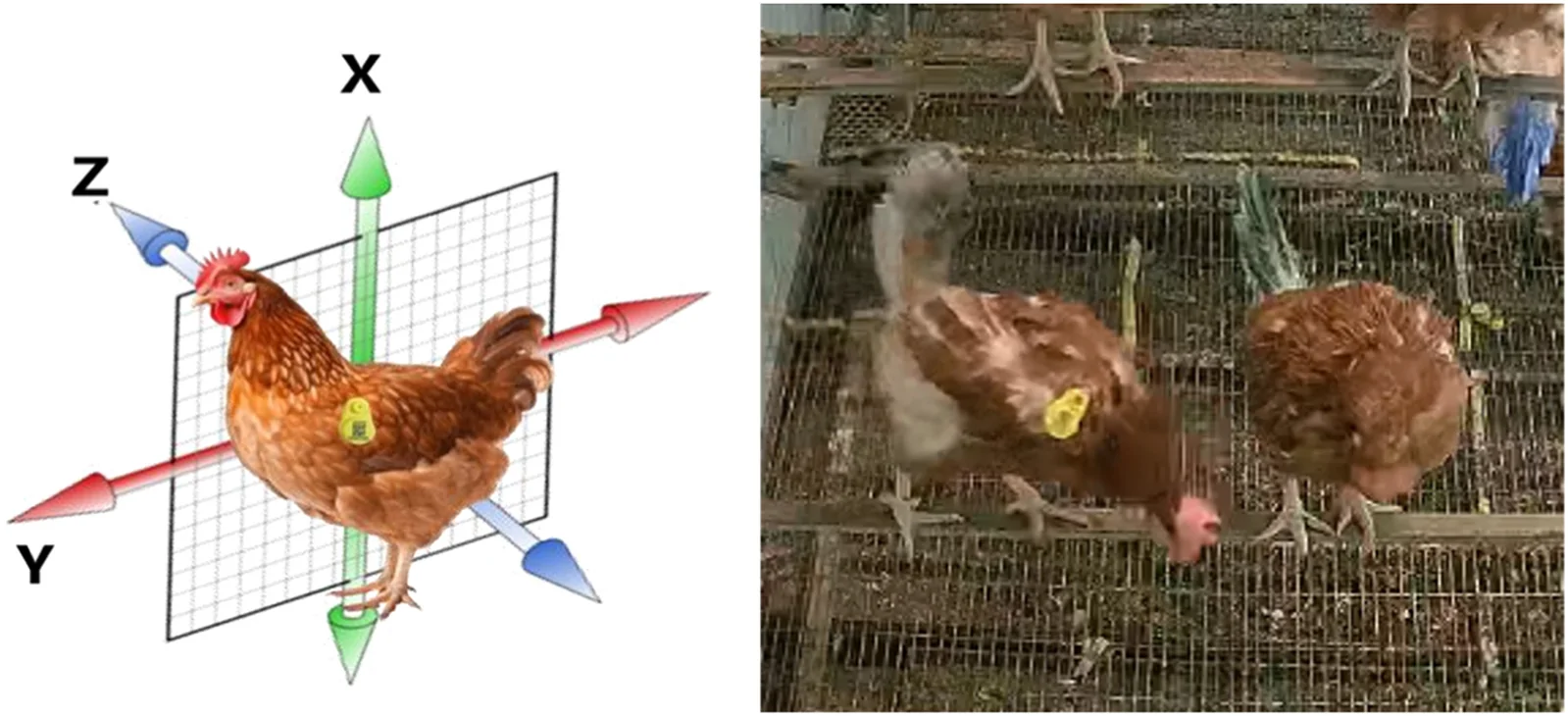
The wing tagged inertial measurement unit.

A total of 25 Hyline laying hens were reared in an aviary system (4.5 × 1.6 × 2.8 m, L × W × H) located at the Experimental Station, China Agricultural University (Fig. 1) at the age of 65 weeks. The system was equipped with two feeder troughs, three nipple drinkers, and a nest box. V-shaped and sloped perch systems were constructed to induce the spatial awareness of laying hens. After a week of acclimation to the housing system, ten birds were randomly selected to wear accelerometers (Fig. 2). To minimize the potential impact of wearable devices on bird behavior, the total weight of the sensor package should generally remain below 3% of the animal’s body weight (Phillips et al., 2003). In the present study, the total weight of the wing-tagged sensor (19.5 g) accounted for approximately 0.99% of the average body weight of the laying hens (approximately 2.0 kg), well below the recommended threshold. In addition, a previous study demonstrated that the effects of wearing a backpack-mounted sensor on laying hens became insignificant after 13-15 days of acclimation (Nie et al., 2022). Therefore, an additional 15-day acclimation period was provided before data collection to ensure that the birds had fully adapted to the device. Therefore, additional 15 days were given to birds to acclimate the sensors before starting the data collection. Four cameras were installed in the housing system to continuously monitoring the activity of laying hens. Feed and water were provided ad libitum. Twice inspections per day were conducted, including egg collection, feeding, and hen inspection. The lighting schedule was 16L:8D, with lights turned on from 0500 h to 2100 h. The temperature was maintained at 16°C - 22°C.

### Data collection

Current accelerometer deployment strategies mainly rely on backpack or chicken harness, which pose considerable challenges for field application and routine maintenance. In this study, a lightweight (48 × 30 × 15 mm, L × W × H) wing-tagged triaxial accelerometer was used. The device is designed to be waterproof and dustproof, and it supports real-time wireless data transmission. Raw acceleration data can be accessed and downloaded via a dedicated platform. Operating at a sampling frequency of 15 Hz, the battery can support the function for over six months. The total weight of the device is 19.5 g, making it suitable for long-term deployment on laying hens with minimal impact on their normal behavior. All experimental procedures were approved by the China Agricultural University Experimental Animal Welfare and Animal Experimentation Ethics Committee.

### Behavioral description and data labelling

**Fig. 3:**
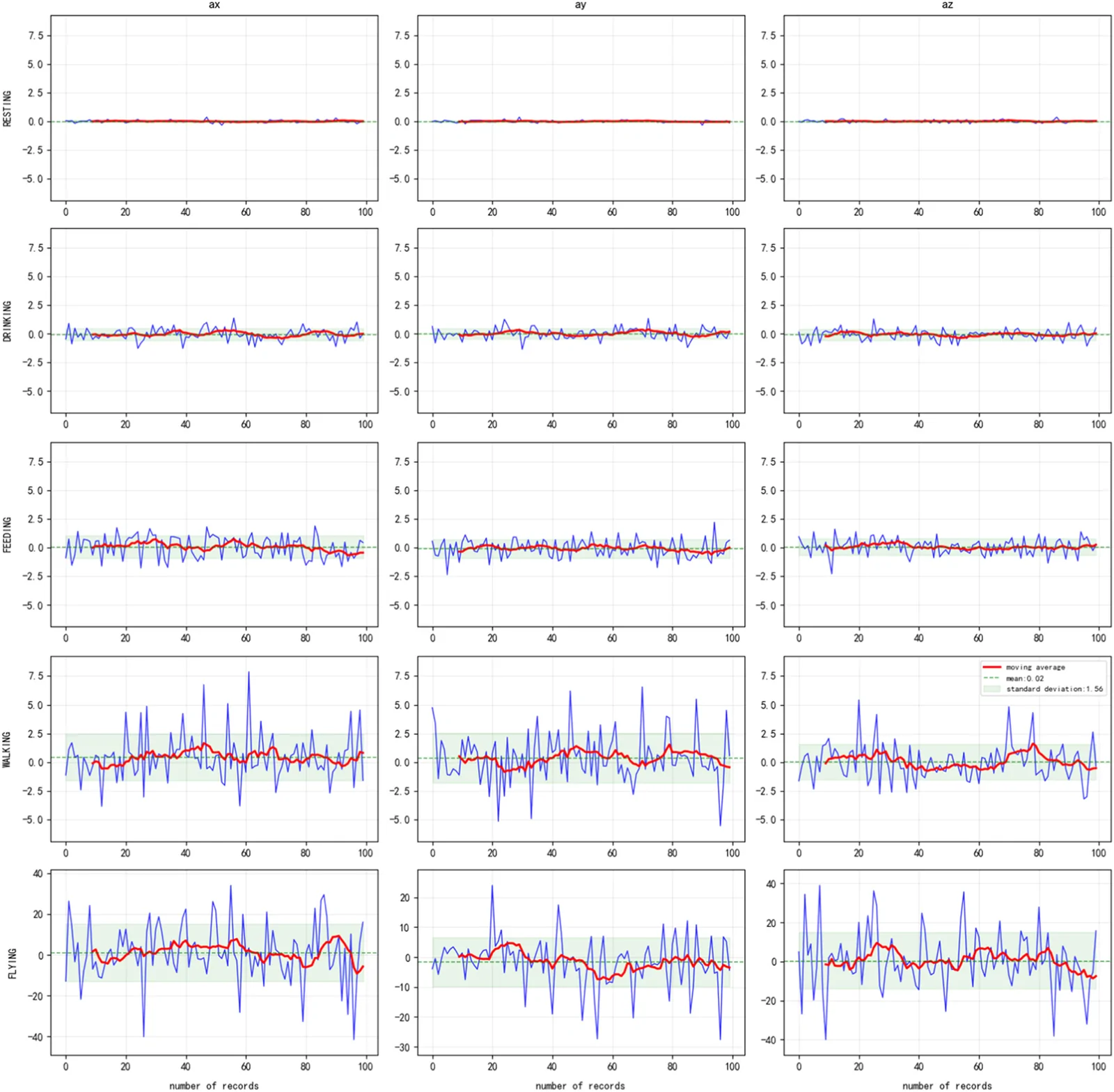
Overview of acceleration signals of different behaviors.

Six behaviors were defined and manually labelled based on recorded videos. The descriptions of behaviors were shown in Table 1, including resting, feeding, drinking, walking, and flying (Fig. 3). Data from ten birds were merged to develop a robust spatial behavior dataset for aviary birds without considering individual differences. The number of labelled records is shown in Table 1. Under sampling was used to create a balanced dataset. Preliminary experiments were conducted to evaluate the necessity of signal denoising. As shown in Figure S1, signal denoising reduced the recognition performance of both representative machine learning and deep learning models. Therefore, raw acceleration signals were used throughout this study.

**Table 1:** Descriptions of different behaviors.

Behavior	Description	Labeled dataset Number of records	Balanced dataset Number of records
Resting	Stand with two feed or sitting on the floor, keep still	80090	23776
Feeding	Pecking at the feed in the feeder trough	24033	23776
Drinking	Keeping the head up and pecking at the nipple drinker	80553	23776
Walking	Moving forward or backward for at least four steps	32192	23776
Flying	Moving from one perch to another, jumping from the floor onto a perch, or jumping directly from the floor to the perch	23776	23776

### Feature extraction

#### Temporal segmentation settings

The selection of sliding window size is critical in acceleration analysis to ensure model performance. An appropriate window size should be sufficient to capture the complete pattern of a given behavior while preserving the temporal dynamics of the signal. The optimal window length largely depends on the duration of individual behavioral events. Evaluated window sizes (1, 2, 3, and 5 s) were selected according to the temporal characteristics of aviary laying hens. Since most behaviors are short-lived and behavioral transitions occur frequently, excessively large windows are prone to include multiple behaviors within a single segment, resulting in feature ambiguity and reduced classification accuracy (Pearce et al., 2024). In addition, different overlapping ratios (0%, 25%, 50%, 70%, and 90%) were investigated to assess their effects on model performance and data representation.

#### Automated feature extraction

Tri-axial acceleration signals were collected and processed to extract informative features for subsequent analysis. To systematically derive discriminative characteristics from the time-series data, the Feature Extraction based on Scalable Hypothesis tests (FRESH) algorithm was employed. This approach enables automated extraction of a large number of candidates features from raw time-series signals, including statistical, temporal, and frequency-domain descriptors. Subsequently, a scalable hypothesis testing framework is applied to evaluate the relevance of each feature with respect to the target variable. Features that pass the significance threshold are retained, while irrelevant or redundant features are discarded. This procedure ensures both computational efficiency and statistical robustness, allowing for effective dimensionality reduction while preserving informative patterns within the tri-axial acceleration data. Approximately 500-1200 features were selected under different sliding window settings (Table 2). Specifically, the features include statistical, temporal, and frequency features. The number of features continuously decreased as the overlapping ratio increased. Additionally, more features can be extracted from larger windows. The selected features were used for training of machine learning models. Summary of effective features under different window configurations is shown in Table 3.

**Table 2:** Feature dimensions under different window configurations.

Overlap ratio (%)	1 s		2 s		3 s		5 s	
	Effective feature	Selected feature	Effective feature	Selected feature	Effective feature	Selected feature	Effective feature	Selected feature
0	1026	829	1107	863	1122	849	1170	864
25	981	794	1098	866	1191	852	1170	867
50	870	709	1053	830	1098	852	1122	836
70	760	608	924	735	1032	802	1098	836
90	675	538	705	556	769	604	885	692

**Table 3:** Summary of effective features under different window configurations.

Window size (s)	Overlap ratio (%)	Frequency	Temporal	Statistical	Other
1	0	0	78	327	621
	25	0	72	327	582
	50	0	60	306	504
	70	0	45	281	434
	90	0	30	270	375
2	0	45	84	327	651
	25	36	84	327	651
	50	27	78	327	621
	70	18	69	306	531
	90	6	33	276	390
3	0	60	84	327	651
	25	54	84	327	651
	50	36	84	327	651
	70	24	75	327	606
	90	9	45	281	434
5	0	60	84	348	678
	25	60	84	348	678
	50	60	84	327	651
	70	36	84	327	651
	90	15	60	306	504

### Model development

In this study, both traditional machine learning algorithms and deep learning models were developed to classify behavioral patterns based on triaxial accelerometer data.

#### Machine learning models

To evaluate the classification performance of the tsfresh‑extracted features, five representative machine learning models were selected, each capturing a distinct learning paradigm, including a linear baseline, an ensemble bagging method, two gradient boosting frameworks, and a kernel‑based method. The SVM was used to construct an optimal hyperplane that maximizes the margin between different behavioral classes in the feature space. K-Nearest Neighbors (KNN) classified samples based on the majority label among the k closest samples in the training dataset according to a distance metric. Extreme Gradient Boosting (XGB) is an ensemble learning method based on gradient boosting that iteratively builds decision trees to minimize prediction errors. Random Forest (RF) is another ensemble learning approach that constructs multiple decision trees using bootstrap sampling and random feature selection, and the final prediction is obtained through majority voting. Decision Tree (DT) is a tree-structured classifier that recursively partitions the feature space based on decision rules derived from input features.

#### Deep learning models

In addition to traditional machine learning algorithms, four deep learning architectures were developed, including one-dimensional Convolutional Neural Network (1D-CNN), Long Short-Term Memory network (LSTM), a hybrid CNN-LSTM model, and a CNN with temporal attention mechanism (CNN-TA).

The 1D-CNN model was applied to automatically learn local temporal patterns from the accelerometer time-series signals. Two convolutional layers with 64 and 128 filters and kernel sizes of 5 and 3 were used to extract hierarchical features from the input sequences. A max-pooling layer was applied after the first convolutional layer to reduce dimensionality and prevent overfitting. The extracted feature maps were then aggregated using a global average pooling layer, followed by a fully connected layer with 64 neurons and a softmax output layer for classification.

The LSTM network was constructed to capture temporal dependencies in the accelerometer data. A single long-short term memory layer with 128 hidden units was used. The output of the LSTM layer was directly fed into a softmax layer for multi-class classification.

To leverage the advantages of both convolutional and recurrent architectures, a hybrid CNN-LSTM model was also implemented. In this architecture, a convolutional layer with 64 filters and a kernel size of 5 was first applied, followed by a max-pooling layer for feature down sampling. The resulting feature maps were then fed into LSTM layer with 128 units to capture long-term temporal dependencies. Finally, the outputs were passed to a softmax classifier. This combined architecture enabled effective learning of both spatial and temporal patterns in the accelerations.

To further enhance temporal feature representation, a CNN model with a temporal attention mechanism was developed (CNN-TA). Thise model shared a similar backbone with the 1D-CNN, consisting of two convolutional layers followed by a max-pooling operation. A temporal attention module was introduced after the convolutional layers to assign adaptive weights to different steps. The attention scores were computed using a fully connected layer with a tanh activation function. The weighted feature maps were then aggregated a global average pooling layer, followed by a dense layer with 64 neurons and a dropout rate of 0.3 to reduce overfitting. Finally, a softmax layer was applied to produce the probability distribution over behavior classes.

### Feature contribution analysis (SHAP)

To interpret the contribution of input features to the model predictions and provide insights into model optimization in the future work, Shapley Additive exPlanations (SHAP) were applied to the optimal machine learning model (XGBoost in this study). Although deep learning models demonstrated competitive performance, we chose the machine learning model for interpretability analysis due to its compatibility with SHAP and the reliability of its explanations. Importantly, the goal of this analysis is not only to interpret a specific model, but to uncover generalizable feature-behavior relationships, therefore, ensuring consistency and theoretical soundness in feature attribution. Furthermore, SHAP enables comprehensive analysis of both individual feature contributions and feature interactions, which are more difficult to obtain reliably from deep learning models. Therefore, the XGBoost model was selected as the basis for interpretability analysis to provide more stable and biologically meaningful insights into behavioral patterns.

SHAP is a game-theoretic approach that quantifies the contribution of each feature to the prediction outcome by computing Shapley values, thereby providing both global and local interpretability. In this study, the TreeExplainer was used. Considering computation efficiency, a subset of the test dataset was randomly sampled for SHAP analysis. Given the multi-class classification setting, SHAP values were computed separately for each behavior and then aggregated by taking the mean absolute value across samples and classes to obtain a unified measure of global feature importance. It was assessed by ranking features based on their mean absolute SHAP values. In addition, SHAP summary plots were generated to visualize the distribution and direction of feature contributions. SHAP interaction values between features were computed using the TreeExplainer to obtain a feature interaction matrix. Based on this matrix, a feature interaction network was constructed, where nodes represent features and edges represent the strength of pairwise interactions. Only the strongest interactions (top percentile) were retained to improve interpretability of the network structure.

### Model performance evaluation

Overall accuracy, precision, recall, and F1 score were calculated to evaluate the performance of instance segmentation model (Equations (1)-4). In order to improve the capability of edge deployment, performance of machine learning models based on different number of features were compared.

(1) $$Overall\;Accuracy=\frac{T_{P}+T_{N}}{T_{P}+T_{N}+F_{P}+F_{N}}$$

(2) $$Precision=\frac{T_{P}}{T_{P}+F_{P}}$$

(3) $$Recall=\frac{T_{P}}{T_{p}+F_{N}}$$

(4) $$F1\;score=2\;\times \frac{Precision\times Recall}{Precision+Recall}$$

Where TP (true positive) is the number of masks that were correctly detected; FN (false negative) is the number of masks that were observed in the image but not detected by the model; FP (false positive) is the number of masks that were detected but not observed in the image; and TN (true negative) is the number of masks that were not detected by the model and not observed in the image.

## Results

1. Overall performance comparison of machine learning models

**Fig. 4:**
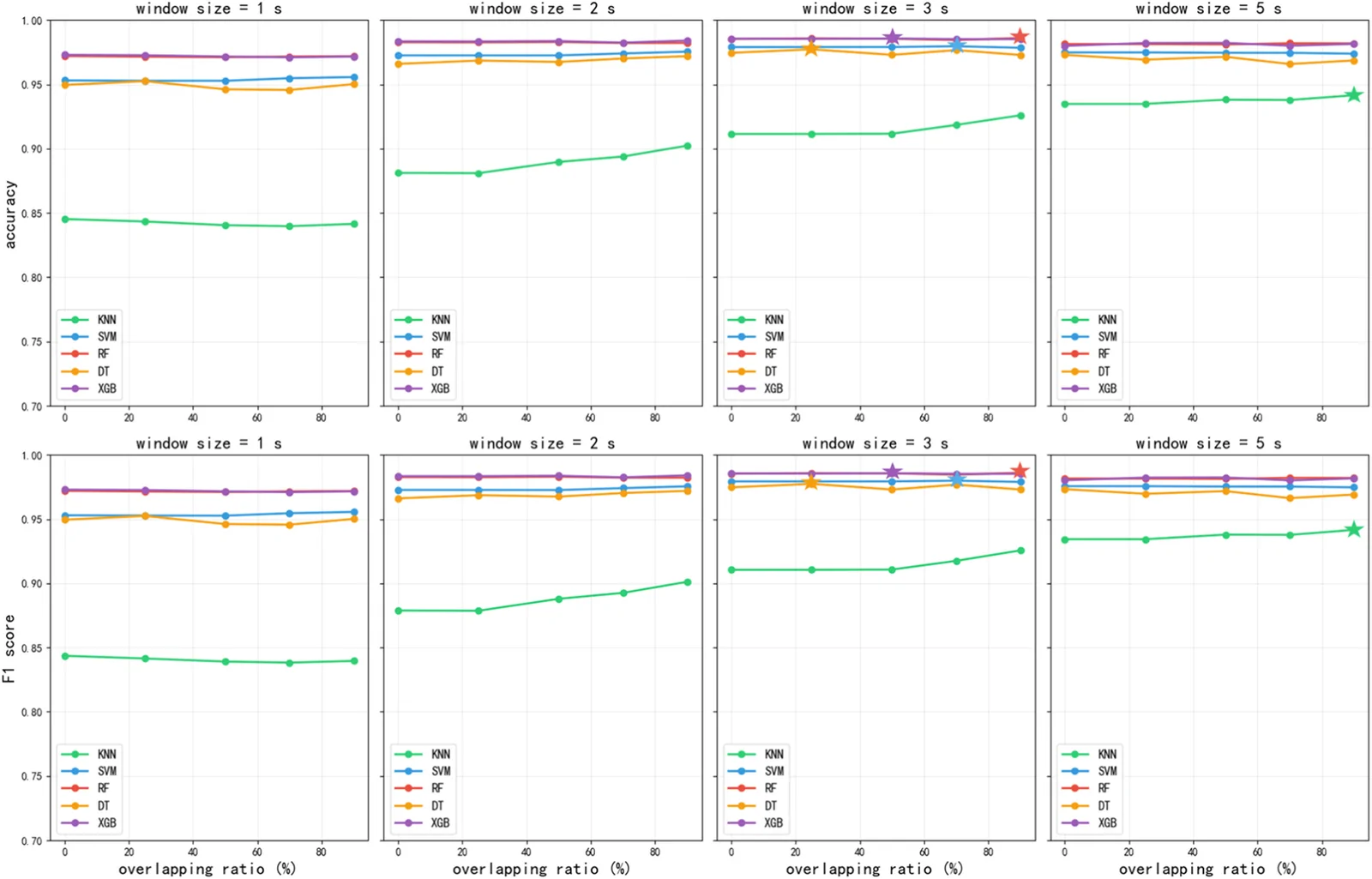
Overall accuracy and F1 score of different machine learning models under different window settings. The optimal setting of each model was labelled with a star.

Fig. 4 shows the overall accuracy and F1 scores of different machine learning models under varying window configurations. For most models, including SVM, DT, RF, and XGB, optimal performance was achieved with a 3-s window, whereas KNN presented a continuously improvement as the window size increased. No consistent trend was observed across different overlap ratios. Generally, variations in model performance were more strongly influenced by size than by overlap ratio. The best F1 scores for KNN, SVM, RF, DT, and XGB were obtained under the following configurations: 5-s window with 90% overlap, 3-s window with 70% overlap, 3-s window with 90% overlap, 3-s window with 25% overlap, and 3-s window with 50% overlap, respectively. Among all combinations, RF yielded the highest performance (accuracy: 0.9860, F1: 0.9861), while KNN reported the lowest (accuracy: 0.8397, F1: 0.8383). Notably, XGB demonstrated more consistent performance across window configurations and was generally comparable to RF, achieving a peak accuracy and F1 score are 0.9856 and 0.9857. The specific values are listed in Table S1.

**Fig. 5:**
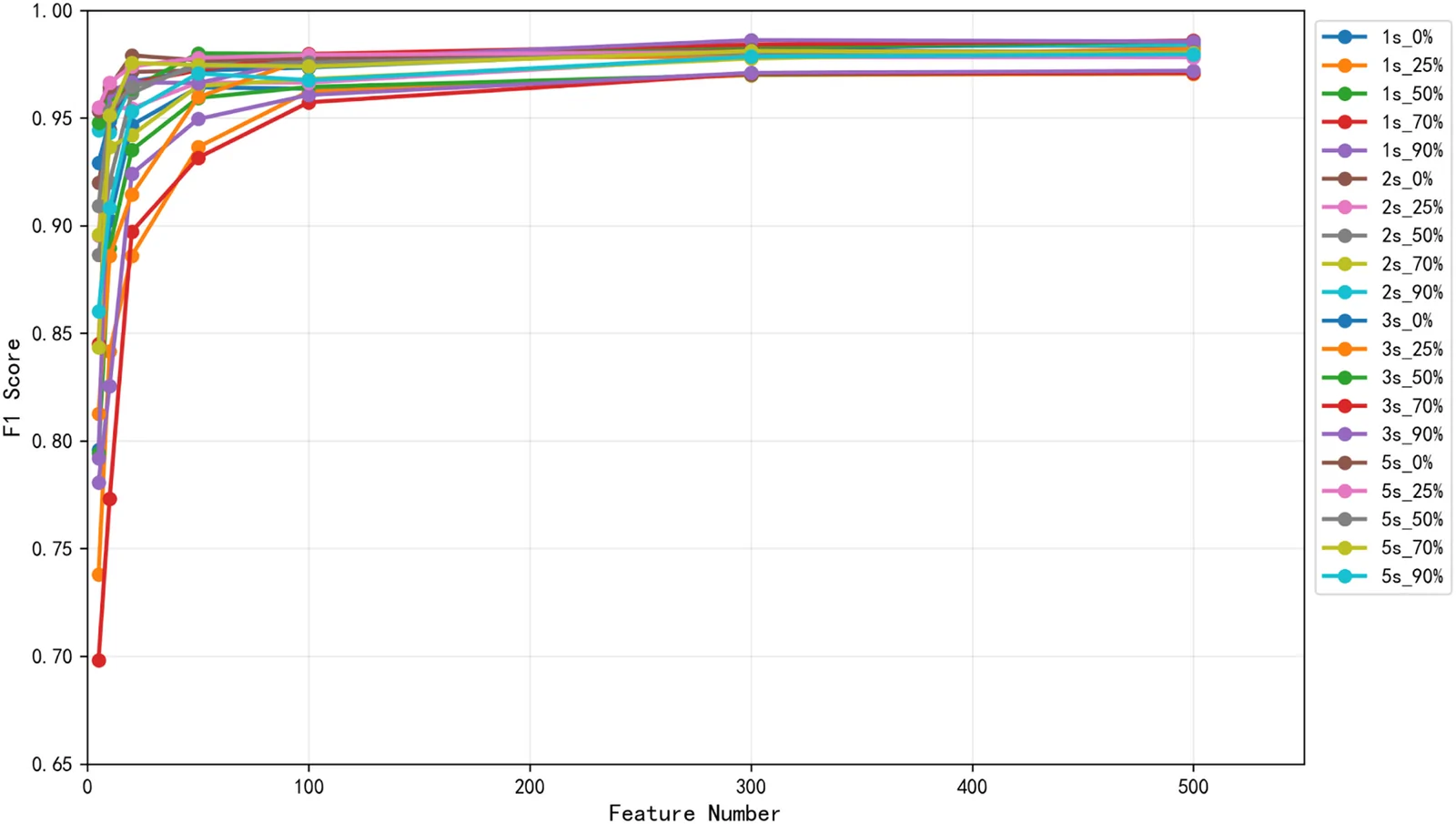
The variation of F1 scores with different feature numbers under each window configuration.

As shown in Fig. 5, both F1 score and overall accuracy exhibited a consistent increasing trend with the number of features. A substantial improvement was observed when the feature number increased from 0 to 100, while performance plateaued beyond 300 features. The optimal performance across window settings was achieved with 300 or 500 features, with accuracy and F1 scores ranging from 0.9706 to 0.9861 and 0.9706-0.9862, respectively. Even with 100 features, acceptable performance was obtained (accuracy: 0.9575-0.9797; and F1: 0.9573-0.9798), including a relatively efficient feature representation.

2. Per-class performance comparison of machine learning models

**Fig. 6:**
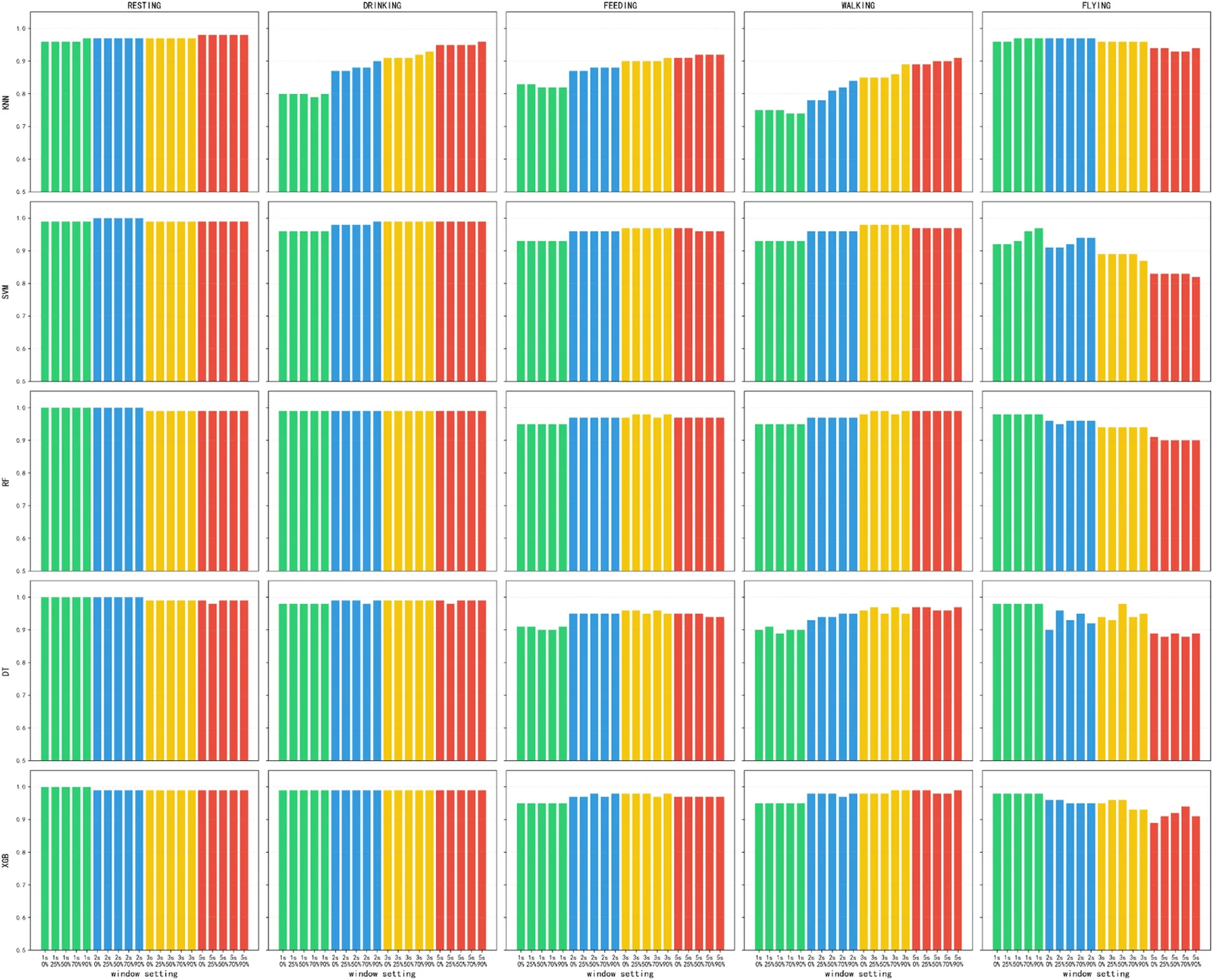
F1 scores of five machine learning models in classifying each behavior under different window settings.

Fig. 6 illustrates the F1 scores for individual behaviors. Resting achieved the highest performance (0.96-1.00), followed by drinking (0.79-0.99), feeding (0.82-0.98), walking (0.74-0.99), and flying (0.82-0.98). For movement-related behaviors, such as drinking, feeding, and walking, larger window sizes (2-5 s) generally yielded better performance compared to 1-s windows, likely due to the richer temporal information captured within the window. In contrast, flying was more accurately identified with smaller windows, reflecting its short-duration and high-intensity characteristics. Window size had minimal impact on resting behavior. The overlap ratio showed limited influence across all behaviors. Similar results were reported by precisions (Figure S2) and recalls (Figure S3). Confusion matrixes of five models under different window settings were shown in Figures S4-S7.

3. Overall performance comparison of deep learning models

**Fig. 7:**
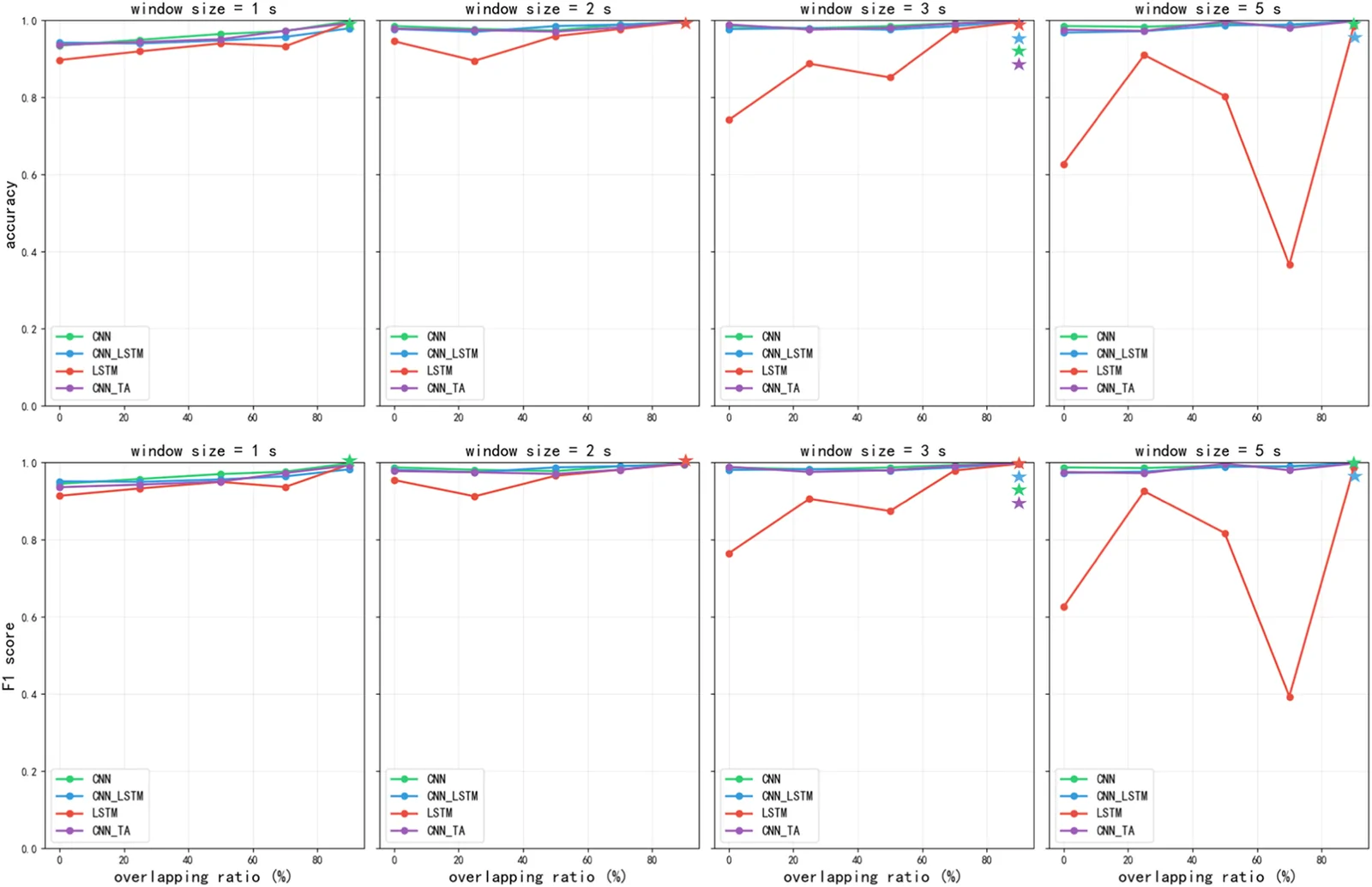
Overall accuracies and F1 scores of four deep learning models under different sliding window settings. The optimal setting of each model was labelled with a star.

Deep learning models demonstrated strong performance in behavior classification (Fig. 7). CNN-based models consistently outperformed LSTM, particularly under larger window sizes. Among them, 1D-CNN achieved the best performance with an accuracy of 0.9981 and 0.9985. In 75% window configurations, 1D-CNN outperformed the CNN-TA architecture. LSTM is more sensitive to both window size and overlap ratio, with accuracy and F1 score varying widely across 0.3652-0.9963 and 0.3931-0.9969. Higher overlap ratios generally improved LSTM performance, especially for larger windows. CNN-based models were less sensitive to overlap ratio, with performance variations within 3% for most window sizes. Overall, a 3-s window with 90% overlap yielded the highest performance across deep learning models. The specific values are listed in Table S2.

4. Per-class performance comparison of deep learning models

**Fig. 8:**
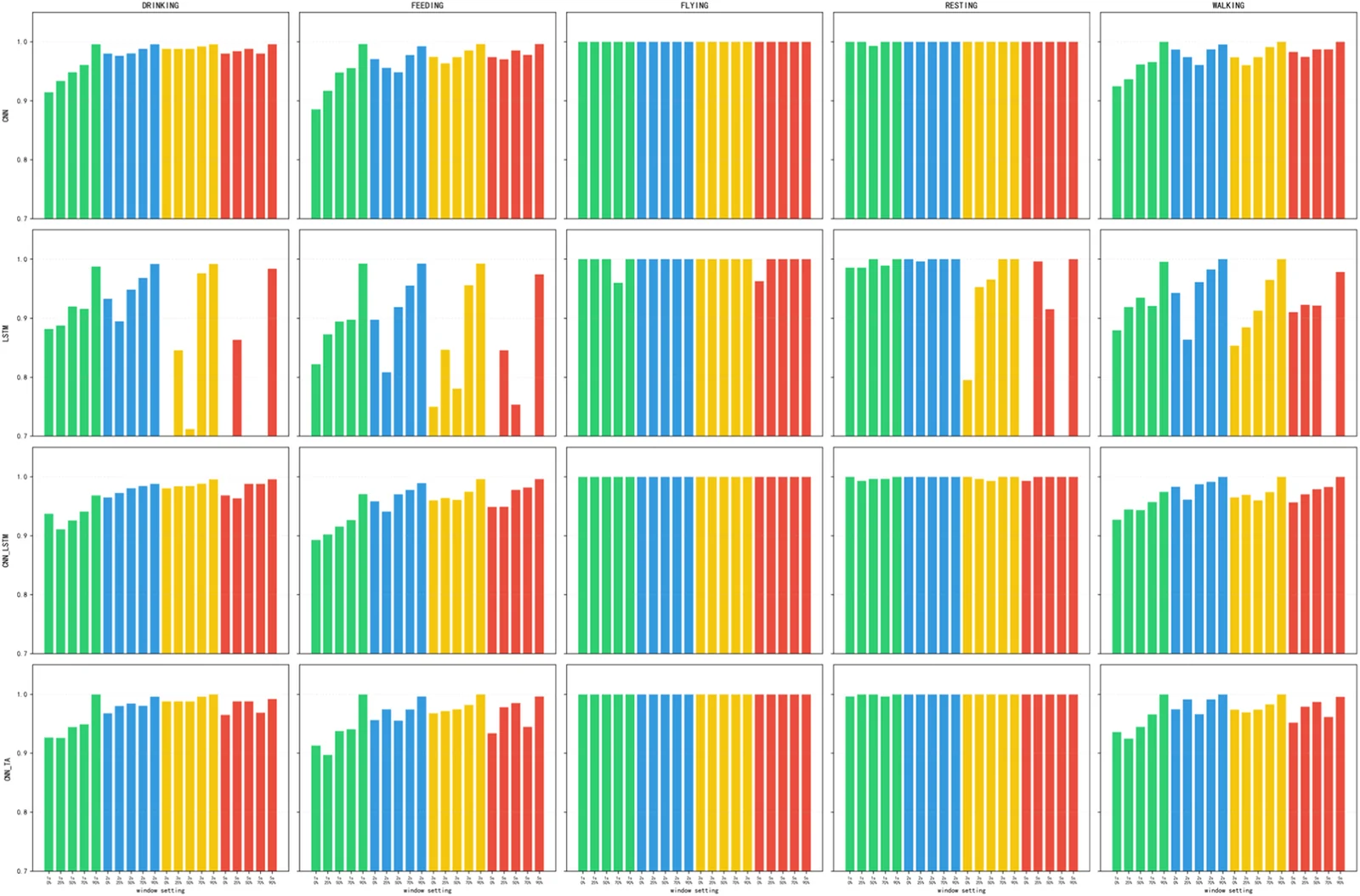
F1 scores of four deep learning models in classifying five behaviors under different sliding window settings.

As shown in Fig. 8, CNN-based models achieved superior performance in classifying drinking, feeding, and walking behaviors compared to LSTM. Window size had limited influence on CNN models, while overlap ratio showed a consistent positive effect on performance. However, although higher overlap ratios improved performance, excessively high overlap may introduce data leakage between training and testing sets., leading to inflated evaluation results. Considering both performance and generalization, the 1D-CNN model with a 3-s window and 50% overlap (F1 = 0.9873) was selected as the optimal configuration. Consistent results were reported by precisions (Figure S8) and recalls (Figure S9). Confusion matrixes of five models under different window settings were shown in Figures S10-S13.

5. SHAP analysis of machine learning models

### Global feature importance

**Fig. 9:**
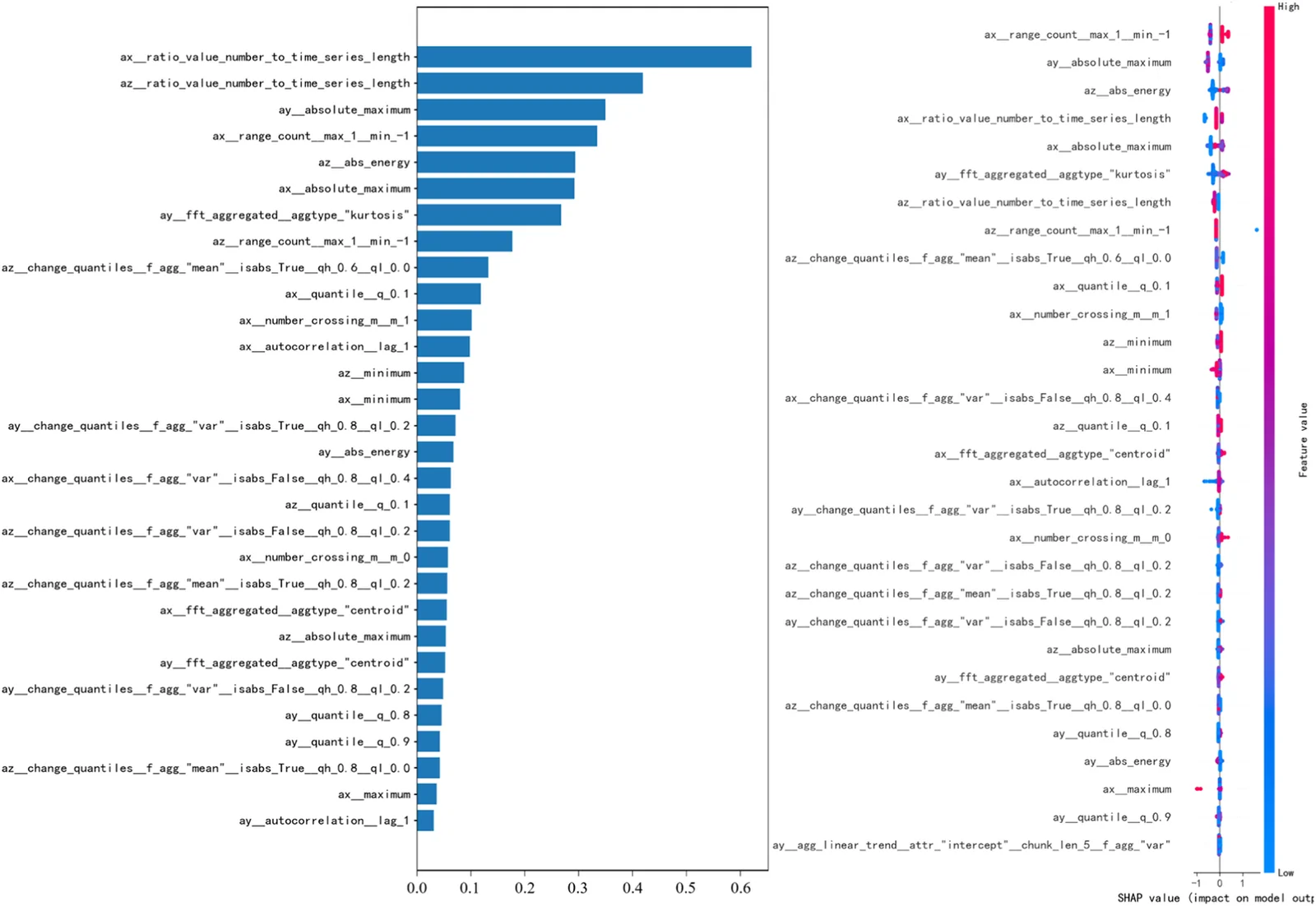
Global feature importance (left) and summary plot (right).

The global feature importance derived from SHAP analysis (Fig. 9) indicates that the model primarily relies on features related to signal variability, amplitude, and energy. Among them, *ratio_value_number_to_time_series_length* is the most influential feature, suggesting that the frequency of signal fluctuations plays a critical role in distinguishing behavioral states. Amplitude-based features, such as absolute maximum and range count, also contribute significantly, reflecting differences in movement intensity. In addition, energy-related features (e.g., *abs_energy*) are particularly important for identifying high-activity behaviors. The SHAP summary plot (Figure XX) further reveals that both high and low feature values contribute differently to the model output, indicating a non-linear relationship between features and behavioral classes.

### Behavior-specific feature contributions

**Fig. 10:**
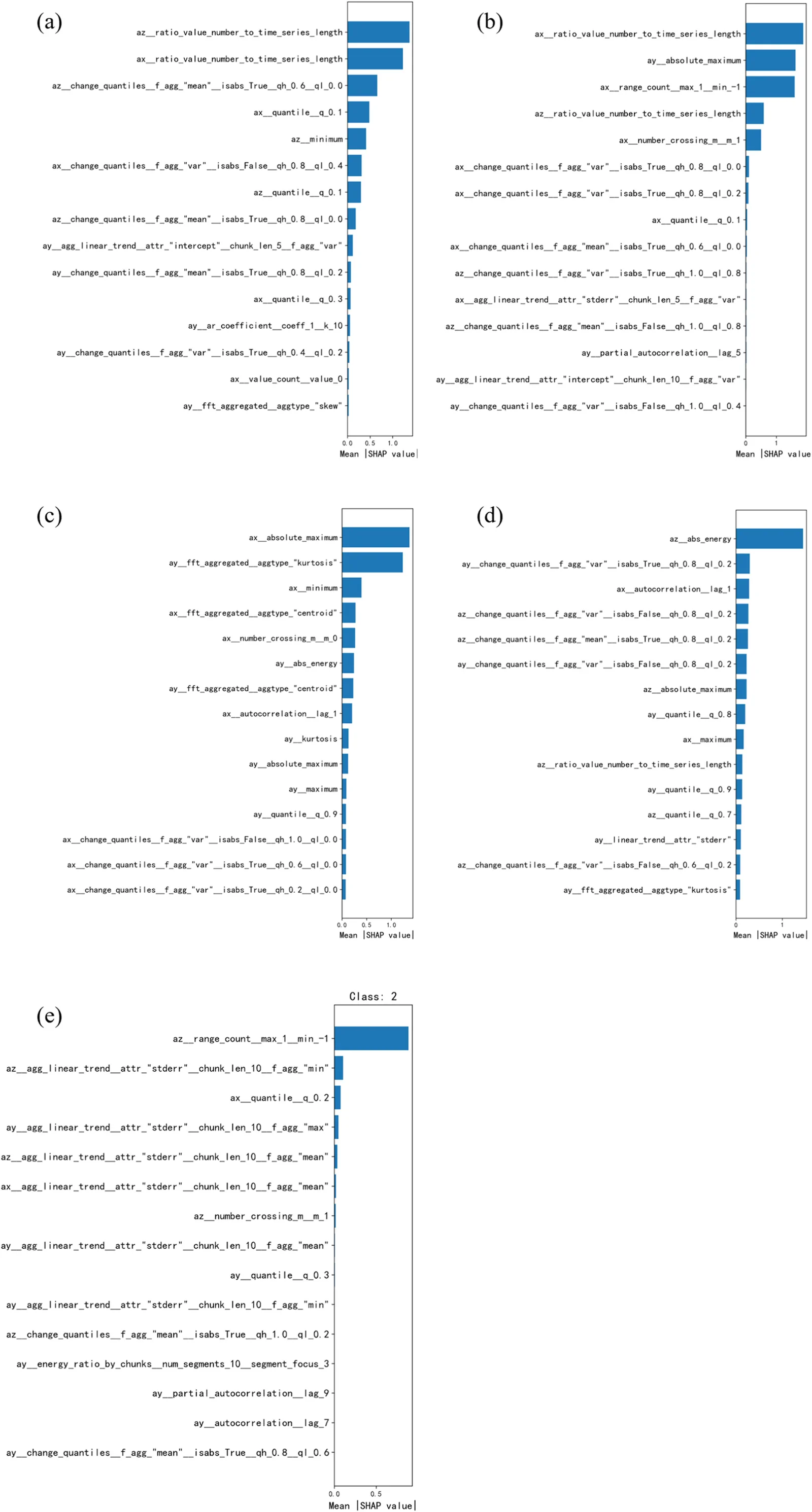
SHAP analysis of resting (a), drinking (b), feeding (c), walking (d), and flying (e).

The class-wise SHAP analysis (Fig. 10) provides insight into how different behaviors are characterized by distinct feature patterns. Flying is primarily associated with high-amplitude and high-energy features, such as *absolute maximum* and *abs_energy*, reflecting the intense and rapid motion generated by wing flapping. In contrast, resting behavior is characterized by low-energy and low-variation features, indicating minimal movement. Walking behavior is distinguished by features related to temporal continuity, such as autocorrelation and frequency-domain descriptors, capturing the rhythmic and sustained nature of locomotion. Feeding and drinking behaviors exhibit similar feature importance patterns, both dominated by low-amplitude signals. Their differentiation relies on higher-order statistical features, such as kurtosis and quantile-based descriptors, suggesting that subtle differences in temporal structure are required to distinguish these behaviors.

### Feature interaction analysis

**Fig. 11:**
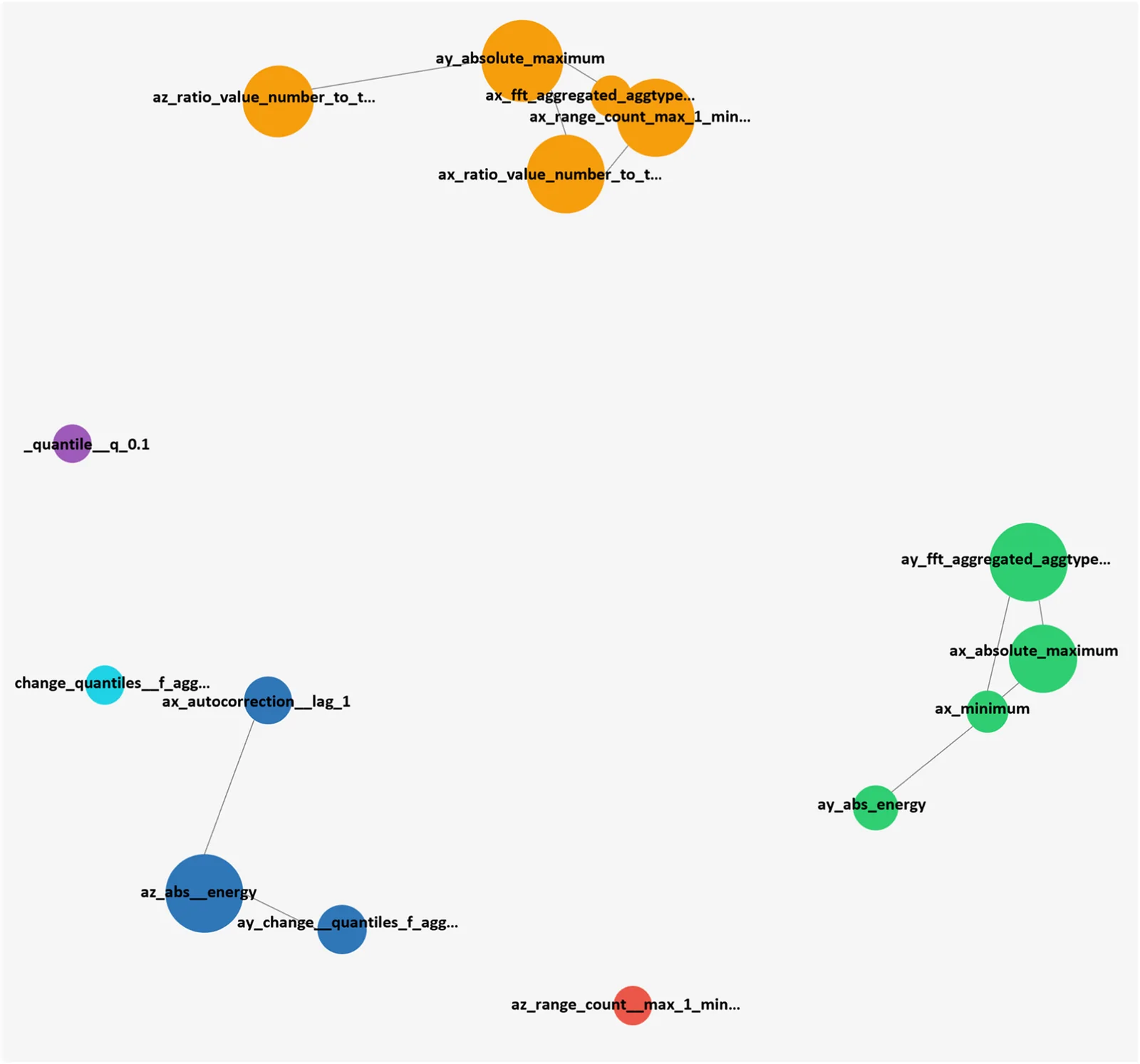
Interaction network of acceleration-based features.

A SHAP-based interaction network was shown in Fig. 11. The network reveals a clear modular structure, indicating that the model relies on groups of interacting features rather than individual variables. Three main clusters can be identified. The first cluster consists of amplitude- and frequency-related features, which show strong interactions and are associated with high-intensity behaviors such as flying. The second cluster includes features related to amplitude and spectral characteristics, forming a chain-like structure that corresponds to moderate and sustained movements, such as walking. The third cluster comprises low-energy and temporal continuity features, which are mainly associated with low-activity behaviors such as resting. Additionally, several features appear as isolated nodes with weak connections, suggesting that they contribute independently to the model prediction. These findings demonstrate that feature synergy plays a critical role in behavior classification, highlighting the importance of considering feature interactions rather than relying solely on individual feature importance.

## Discussion

### Temporal segmentation

This study systematically evaluated the effects of sliding window sizes and overlap ratios on the recognition of laying hen behaviors. The results demonstrated that window size plays a more critical role than overlap ratio in determining model performance, particularly for machine learning models. Larger windows capture more temporal patterns, which is beneficial for identifying sustained behaviors such as feeding and walking. In contrast, short-duration behaviors such as flying are better captured by smaller windows, highlighting the importance of matching temporal resolution to behavioral characteristic. Many studies on broiler behaviors have discussed the effects of window configuration on model performance with the window size ranged from 0.5 s to 30 s (Pearce et al., 2024). Zhang et al. (2024) compared the different window length (2, 5, 8, and 12 s) in classifying broiler behaviors. The results indicate the 8-s window yielded the optimal performance. Yang et al. (2021) found that 7-s window performed better in identifying resting of broiler, while 20-s window outperformed other configurations in classifying feeding and drinking. Much smaller window sizes (0.5 and 1 s) were studied in aviary laying hens and found 1-s window yielded better performance (Yang et al., 2025). As no larger windows were compared, it is unsure whether the performance can be further improved under larger sliding windows. Behaviors of aviary laying hens are quite different from those of broiler due to their higher activity frequency and more frequent behavioral transitions. Our findings are consistent with these observations, as movement-related behaviors (feeding, drinking, and walking) benefited from larger windows, whereas flying was better recognized with shorter windows. This is probably because larger window size may cover the variation pattern of flying and compromise the identification performance. These findings suggest that optimal window configuration should be behavior-dependent and determined according to the temporal continuity and dynamic characteristics of target activities, rather than being universally fixed across recognition tasks. In addition, the temporal segmentation parameters identified in this study may theoretically provide broad reference value for accelerometer-based behavior recognition in laying hens, as behavioral dynamics during the production stage are relatively stable. Unlike broilers, which typically undergo rapid body weight increases within a few weeks of growth, laying hens generally maintain relatively stable body weight and locomotion characteristics throughout the laying period. However, variations in age, breed, housing conditions, and locomotion ability may still affect the optimal parameter configuration to some extent.

Although higher overlap ratios consistently improved model performance, this effect should be interpreted with caution. High overlap introduces substantial redundancy between adjacent samples, increasing the similarity between training and testing data. This leads to a form of data leakage, where the model may implicitly learn highly similar temporal patterns appearing in both training and testing sets, thereby inflating evaluation metrics without improving true generalization ability. Therefore, a moderate overlap ratio (e.g., 50%) provides a better balance between sample size and generalization ability, which have been widely adopted in previous studies (Pearce et al., 2024; Yang et al., 2025).

### Feature dimensionality

Feature engineering has substantial impact on accelerometer-based behavior recognition using machine learning models. A variety of handcrafted features have been explored in previous studies. Commonly used features include statistical descriptors (e.g., mean, standard deviation, skewness, and kurtosis), amplitude-related features (e.g., maximum, minimum, and range), energy-related features (e.g., signal magnitude area and absolute energy), and frequency-domain features obtained through Fourier or spectral analysis. Most previous studies rely on a relatively limited set of predefined features, which may not fully represent the complexity of behavioral patterns, particularly for subtle or similar activities such as feeding and drinking. In contrast, the feature extraction approach used in this study, based on tsfresh, systematically generates a large number of features across multiple domains, including higher-order statistics and temporal structure descriptors. This comprehensive representation enables the model to capture fine-grained differences between behaviors. At the same time, it also suggests that some informative features may have been overlooked in earlier studies due to manual feature selection.

Although the features generated by tsfresh can provide a richer representation of behavioral patterns, enabling models to effectively distinguish subtle differences between behaviors, the use of high-dimensional feature sets also raises important considerations for practical deployment. It introduces increased computational cost in both feature extraction and model inference. This may limit its on field real-time application. Therefore, a trade-off exists between model accuracy and computational efficiency. It was observed that acceptable performance was still achieved in this study using only 100 features, while performance gains became marginal beyond 300 features, indicating substantial feature redundancy. This observation suggests that compact yet informative feature subsets may be preferable for real-world deployment of accelerometer-based monitoring systems.

### Model architecture

Most previous studies on accelerometer-based poultry behavior recognition have focused on machine learning models. Zhang et al. (2024) achieved an overall accuracy of 93.9% for broiler behavior classification based on RF model. Other approaches such as KNN, SVM, Naive Bayes, and DT, reported the accuracies ranging from 62% to 99%, depending on sensor configuration, feature engineering, and model complexity (Yang et al., 2025). Consistent with previous findings, resting behavior was easier to identify, whereas feeding and drinking are difficult to be differentiated. Overall, both machine learning and deep learning models achieved strong performance in this study. The improvement is likely attributed to the comprehensive feature extraction strategy, which captures statistical, temporal, and frequency-domain characteristics of accelerometer signals.

Among the evaluated models, the 1D-CNN achieved the best overall performance, outperforming more complex architectures such as CNN-LSTM and CNN-TA. This result suggests that increasing architecture complexity does not necessarily improve accelerometer-based behavior recognition. One possible explanation is that the relatively short sliding windows (1-5 s) used in this study primarily contain local temporal patterns, which can already be effectively captured by convolutional operations (Alzubaidi et al., 2021). Consequently, recurrent and attention-based modules may introduce additional complexity without providing substantial benefits, while also increasing the risk of overfitting. These findings indicate that model architecture should be matched to the temporal characteristics and intrinsic complexity of the target behaviors. For short- and medium-duration poultry behaviors, lightweight convolutional architectures may provide a more suitable balance between recognition accuracy, robustness, and computational efficiency.

From a practical perspective, the 1D-CNN shows strong potential for edge applications due to its end-to-end structure and minimal feature engineering requirements. In contrast, the XGBoost, despite relying on handcrafted high-dimensional features, offers greater inutterability and enables a more comprehensive understanding of the behavioral mechanisms underlying model predictions. Therefore, the choice between machine learning and deep learning approaches should consider not only predictive performance, but also interpretability, computational efficiency, and deployment requirements.

### Model interpretability

The SHAP results provide important insights into the behavioral mechanisms underlying model predictions. Global feature importance indicates that variability, amplitude, and energy are the primary factors distinguishing different behaviors. These feature categories are closely associated with movement intensity, motion stability, and temporal dynamics, suggesting that they may serve as generally informative descriptors for accelerometer-based animal behavior recognition. Behavior-based specific analysis further reveals that different activities are characterized by distinct signal patterns. For example, flying is associated with high-energy bursts, while resting is characterized by minimal signal variation. Walking behavior is identified by rhythmic temporal patterns, consistent with its locomotion nature. Feeding and drinking behaviors both involve head movements with relatively small amplitudes, making them difficult to distinguish based on intensity alone (Zhang et al., 2024). The model therefore relies on higher-order statistical features to capture subtle differences in motion patterns. Importantly, the feature interaction analysis revealed that behavior classification is not driven by individual features alone, but rather by the synergistic effects of multiple feature groups. The modular structure observed in the interaction network suggests that different groups of features correspond to different behavioral states. This finding emphasizes the importance of considering feature interactions in time-series behavior analysis. It may explain why models capable of learning nonlinear feature relationships, such as XGBoost and CNN-based architectures, achieved better performance in this study. In addition, the multidimensional interaction patterns may provide useful insights for detecting subtle behavioral abnormalities associated with health or welfare issues, which are often difficult to identify using single-feature indicators.

Overall, this study demonstrates that accelerometer-based poultry behavior recognition should not rely solely on maximizing model complexity or feature dimensionality. Instead, effective behavior recognition depends on balancing temporal segmentation, feature representation, model interpretability, and deployment feasibility. Only wing-tagged accelerometers were investigated in this study, the feasibility of the proposed framework on other sensor placement strategies require further validation. Future studies should explore adaptive temporal segmentation strategies that dynamically optimized window configurations based on behavioral characteristics.

### Limitations and future perspectives

Although the proposed models achieved promising performance, several limitations should be acknowledged. First, feeding and drinking remain relatively difficult to distinguish because both behaviors are characterized by subtle head movements with similar acceleration characteristics. Future studies may improve the discrimination of these highly similar behaviors by incorporating longer temporal contexts, behavioral transition information, or more discriminative representation learning strategies. Second, although the proposed framework demonstrated promising performance under experimental aviary systems, practical implementation will require further improvements in sensor durability, battery life, data transmission reliability, and the cost-effectiveness of long-term monitoring in commercial aviary systems. Finally, extending the models toward continuous welfare monitoring by integrating long-term behavioral dynamics, behavioral transition patterns, and physiological indicators represents an important direction for future research. Such developments could facilitate continuous welfare assessment, early detection of health abnormalities, and more comprehensive decision support for precision management in commercial aviary systems.

## Conclusions

This study proposed a comprehensive framework for accelerometer-based behavior recognition in aviary laying hens by integrating temporal segmentation, feature engineering, model comparison, parameter optimization, and model interpretation. Machine learning and deep learning approaches were systematically evaluated under multiple sliding window configurations. Among the evaluated temporal segmentation strategies, window size had a greater impact on identification performance than overlap ratio, and a 3-s window with 50% overlap provided the best overall performance. High-dimensional features extraction significantly improved classification performance, and performance gains was minimal beyond 100 features, suggesting the existence of feature redundancy. Among evaluated models, the lightweight 1D-CNN achieved the best performance (accuracy = 0.985, F1 score = 0.985), whereas more complex architectures did not consistently provide addition benefits. SHAP analysis further revealed that variability amplitude-, and energy-related features contributed most substantially to model predictions in poultry behavior classification. Overall, this study provides practical methodology guidance for developing accurate and interpretable accelerometer-based behavior recognition in precision poultry farming.

## Author Contribution

**Xiao Yang** and **Luwei Nie** conceived and designed the study. **Qunpeng Niu, Xiaoqiang Li**, and **Juncheng Ma** performed the data acquisition, analysis, and model development. Chaoyuan Wang provided critical resources and supervised the project. Xiao Yang drafted the manuscript, and all authors reviewed and edited the manuscript critically for crucial intellectual content. All authors have read and approved the final version of the manuscript and agree to be personally and publicly accountable for all aspects of the work, including ensuring that any questions regarding its accuracy or integrity are thoroughly investigated and resolved.

## Disclosures

The authors declare that they have no known competing financial interests or personal relationships that could have appeared to influence the work reported in this paper.

## Appendix Supplementary materials

Image, application 1

## Footnotes

Supplementary material associated with this article can be found, in the online version, at doi:10.1016/j.psj.2026.107539.
