# Supplementary material for: Feature engineering, parameter optimization, and interpretable modeling for accelerometer-based behavior recognition in aviary laying hens
Source: Poult Sci. 2026 Jul 30;105(11):107539. doi: 10.1016/j.psj.2026.107539 (PMC13572173; doi:10.1016/j.psj.2026.107539)
Supplement: Supplementary file 1 [file mmc1.zip › Supplementary figure caption.docx]

Figure S1 Performance comparison of XGB (first row) and 1D-CNN (second row) before and after denoising. Four columns refer to overall accuracy, F1 score, precision, and recall, respectively.

Figure S2 Precisions of different machine learning models in classifying each behavior under different window sizes

Figure S3 Recalls of different machine learning models in classifying each behavior under different window sizes

Figure S4 Confusion matrixes of different machine learning models under 1-s window

Figure S5 Confusion matrixes of different machine learning models under 2-s window

Figure S6 Confusion matrixes of different machine learning models under 3-s window

Figure S7 Confusion matrixes of different machine learning models under 5-s window

Figure S8 Precisions of different deep learning models in classifying each behavior under different window sizes

Figure S9 Recalls of different deep learning models in classifying each behavior under different window sizes

Figure S10 Confusion matrixes of different deep learning models under 1-s window

Figure S11 Confusion matrixes of different deep learning models under 2-s window

Figure S12 Confusion matrixes of different deep learning models under 3-s window

Figure S13 Confusion matrixes of different deep learning models under 5-s window
