# Supplementary material for: Feature engineering, parameter optimization, and interpretable modeling for accelerometer-based behavior recognition in aviary laying hens
Source: Poult Sci. 2026 Jul 30;105(11):107539. doi: 10.1016/j.psj.2026.107539 (PMC13572173; doi:10.1016/j.psj.2026.107539)
Supplement: Supplementary file 1 [file mmc1.zip › Supplementary table.docx]

Table S1 performance of different machine learning models under different window settings

| Overlap  ratio | Model | 1 s | | 2 s | | 3 s | | 5 s | |
| --- | --- | --- | --- | --- | --- | --- | --- | --- | --- |
|  |  | Accuracy | F1 | Accuracy | F1 | Accuracy | F1 | Accuracy | F1 |
| 0 | KNN | 0.8452 | 0.8436 | 0.8812 | 0.8789 | 0.9114 | 0.9105 | 0.9347 | 0.9344 |
|  | SVM | 0.9531 | 0.9530 | 0.9725 | 0.9727 | 0.9789 | 0.9793 | 0.9748 | 0.9756 |
|  | RF | 0.9720 | 0.9720 | 0.9827 | 0.9827 | 0.9852 | 0.9853 | 0.9813 | 0.9816 |
|  | DT | 0.9496 | 0.9496 | 0.9659 | 0.9661 | 0.9745 | 0.9747 | 0.9729 | 0.9733 |
|  | XGB | 0.9729 | 0.9729 | 0.9834 | 0.9834 | 0.9854 | 0.9855 | 0.9799 | 0.9802 |
| 25 | KNN | 0.8433 | 0.8415 | 0.8810 | 0.8787 | 0.9114 | 0.9105 | 0.9347 | 0.9344 |
|  | SVM | 0.9528 | 0.9528 | 0.9725 | 0.9727 | 0.9790 | 0.9793 | 0.9748 | 0.9756 |
|  | RF | 0.9715 | 0.9715 | 0.9826 | 0.9826 | 0.9857 | 0.9858 | 0.9813 | 0.9815 |
|  | DT | 0.9525 | 0.9525 | 0.9685 | 0.9686 | 0.9772 | 0.9774 | 0.9692 | 0.9697 |
|  | XGB | 0.9726 | 0.9726 | 0.9833 | 0.9834 | 0.9853 | 0.9854 | 0.9821 | 0.9823 |
| 50 | KNN | 0.8405 | 0.8391 | 0.8897 | 0.8880 | 0.9116 | 0.9107 | 0.9381 | 0.9380 |
|  | SVM | 0.9528 | 0.9527 | 0.9725 | 0.9726 | 0.9790 | 0.9793 | 0.9745 | 0.9753 |
|  | RF | 0.9711 | 0.9711 | 0.9829 | 0.9830 | 0.9854 | 0.9856 | 0.9810 | 0.9813 |
|  | DT | 0.9462 | 0.9462 | 0.9674 | 0.9675 | 0.9729 | 0.9730 | 0.9714 | 0.9718 |
|  | XGB | 0.9716 | 0.9716 | 0.9836 | 0.9837 | 0.9856 | 0.9857 | 0.9822 | 0.9824 |
| 70 | KNN | 0.8397 | 0.8383 | 0.8939 | 0.8926 | 0.9184 | 0.9175 | 0.9379 | 0.9378 |
|  | SVM | 0.9548 | 0.9546 | 0.9740 | 0.9741 | 0.9796 | 0.9799 | 0.9745 | 0.9753 |
|  | RF | 0.9716 | 0.9716 | 0.9821 | 0.9822 | 0.9844 | 0.9845 | 0.9819 | 0.9821 |
|  | DT | 0.9457 | 0.9457 | 0.9701 | 0.9703 | 0.9766 | 0.9768 | 0.9658 | 0.9663 |
|  | XGB | 0.9710 | 0.9710 | 0.9824 | 0.9825 | 0.9851 | 0.9852 | 0.9800 | 0.9801 |
| 90 | KNN | 0.8415 | 0.8396 | 0.9023 | 0.9012 | 0.9259 | 0.9256 | 0.9416 | 0.9417 |
|  | SVM | 0.9557 | 0.9556 | 0.9754 | 0.9755 | 0.9784 | 0.9789 | 0.9738 | 0.9747 |
|  | RF | 0.9719 | 0.9719 | 0.9822 | 0.9822 | 0.9860 | 0.9861 | 0.9818 | 0.9820 |
|  | DT | 0.9502 | 0.9502 | 0.9719 | 0.9720 | 0.9728 | 0.9730 | 0.9686 | 0.9691 |
|  | XGB | 0.9716 | 0.9716 | 0.9840 | 0.9840 | 0.9851 | 0.9852 | 0.9814 | 0.9817 |

Table S2 Overall accuracies and F1 scores of five deep learning models under different sliding window settings

| Overlap ratio | Model | 1 s | | 2 s | | 3 s | | 5 s | |
| --- | --- | --- | --- | --- | --- | --- | --- | --- | --- |
|  |  | Accuracy | F1 | Accuracy | F1 | Accuracy | F1 | Accuracy | F1 |
| 0 | 1D-CNN | 0.9346 | 0.9450 | 0.9850 | 0.9876 | 0.9850 | 0.9873 | 0.9850 | 0.9875 |
|  | LSTM | 0.8972 | 0.9141 | 0.9458 | 0.9549 | 0.7416 | 0.7649 | 0.6273 | 0.6254 |
|  | CNN_LSTM | 0.9421 | 0.9515 | 0.9776 | 0.9813 | 0.9775 | 0.9811 | 0.9682 | 0.9733 |
|  | CNN_TA | 0.9366 | 0.9363 | 0.9780 | 0.9781 | 0.9890 | 0.9890 | 0.9756 | 0.9755 |
| 25 | 1D-CNN | 0.9495 | 0.9576 | 0.9776 | 0.9813 | 0.9794 | 0.9825 | 0.9831 | 0.9858 |
|  | LSTM | 0.9196 | 0.9332 | 0.8953 | 0.9127 | 0.8876 | 0.9061 | 0.9101 | 0.9257 |
|  | CNN_LSTM | 0.9402 | 0.9502 | 0.9701 | 0.9750 | 0.9794 | 0.9828 | 0.9719 | 0.9766 |
|  | CNN_TA | 0.9436 | 0.9431 | 0.9750 | 0.9749 | 0.9759 | 0.9758 | 0.9727 | 0.9727 |
| 50 | 1D-CNN | 0.9645 | 0.9703 | 0.9738 | 0.9780 | 0.9850 | 0.9873 | 0.9906 | 0.9921 |
|  | LSTM | 0.9402 | 0.9500 | 0.9589 | 0.9658 | 0.8521 | 0.8744 | 0.8034 | 0.8171 |
|  | CNN_LSTM | 0.9477 | 0.9564 | 0.9850 | 0.9876 | 0.9757 | 0.9796 | 0.9869 | 0.9889 |
|  | CNN_TA | 0.9510 | 0.9509 | 0.9707 | 0.9706 | 0.9803 | 0.9803 | 0.9970 | 0.9969 |
| 70 | 1D-CNN | 0.9720 | 0.9765 | 0.9888 | 0.9906 | 0.9925 | 0.9938 | 0.9869 | 0.9891 |
|  | LSTM | 0.9327 | 0.9368 | 0.9776 | 0.9814 | 0.9757 | 0.9796 | 0.3652 | 0.3931 |
|  | CNN_LSTM | 0.9570 | 0.9643 | 0.9888 | 0.9907 | 0.9850 | 0.9873 | 0.9888 | 0.9905 |
|  | CNN_TA | 0.9729 | 0.9728 | 0.9817 | 0.9816 | 0.9915 | 0.9915 | 0.9802 | 0.9803 |
| 90 | 1D-CNN | 0.9981 | 0.9985 | 0.9963 | 0.9969 | 0.9981 | 0.9985 | 0.9981 | 0.9985 |
|  | LSTM | 0.9944 | 0.9953 | 0.9963 | 0.9969 | 0.9963 | 0.9969 | 0.9850 | 0.9874 |
|  | CNN_LSTM | 0.9794 | 0.9827 | 0.9944 | 0.9954 | 0.9981 | 0.9985 | 0.9981 | 0.9985 |
|  | CNN_TA | 0.9929 | 0.9929 | 0.9985 | 0.9985 | 0.9990 | 0.9990 | 0.9977 | 0.9977 |
